# The complete chloroplast genome of *Corispermum patelliforme* (Amaranthaceae): genome characterization and phylogenetic consideration

**DOI:** 10.1080/23802359.2025.2561080

**Published:** 2025-09-18

**Authors:** Zhen Yang, Jinju Zhao, Wenqin Li, Yangjie Chen, Chaopan Zhang, Hongqiang Lin

**Affiliations:** ^a^ School of Brewing Engineering, Moutai Institute, Renhuai, Guizhou, P. R. China; ^b^ School of Biological Science & Engineering, North Minzu University, Yinchuan, Ningxia, P. R. China; ^c^ Sichuan Wolong National Natural Reserve Administration Bureau, Wenchuan, Sichuan, P. R. China

**Keywords:** *Corispermum patelliforme*, Amaranthaceae, chloroplast genome, phylogenetic analysis

## Abstract

*Corispermum patelliforme* belongs to the family Amaranthaceae and plays a critical role in ecological systems. In this study, we assembled the chloroplast genomes of *C. patelliforme* for the first time. The result revealing a 150,614 bp circular structure with typical quadripartite organization: 84,222 bp LSC, 19,740 bp SSC, and two 46,652 bp IRs. The genome contains 128 genes and has 36.5% GC content. Phylogenetic analyses (ML/BI) of 15 Amaranthaceae species indicated that *C. patelliforme* and *C. pamiricum* forming a monophyletic group, confirming their close relationship. This study advances understanding of chloroplast evolution in Amaranthaceae.

## 1. Introduction

*Corispermum patelliforme* Iljin 1929 (Iljin 1929), an annual species in the genus *Corispermum* L. (Amaranthaceae Juss.), is distributed to arid regions of northern China, particularly distributed across Gansu, Qinghai, Ningxia, and Inner Mongolia (Liu 1985; Wu and Raven 2003; Yao et al. 2019). It thrives in desert, semi-desert, and sandy grassland ecosystems (Liu et al. 2010; Gao et al. 2021; Bao et al. 2024). Recognized as a pioneer species in sandy arid environments, this plant plays a critical role in stabilizing mobile dunes and initiating ecological succession. Additionally, *C. patelliforme* serves as a vital early-spring forage resource for grazing livestock such as cattle and sheep due to its high nutritional value in desert habitats (Bao et al. 2024; Sukhorukov et al. 2024). Previous studies have focused on the seed germination and medicinal value of *C. patelliforme* (Liu et al. 2010; Zhou et al. 2019). Despite its economic and ecological significance, much remains unknown about *C. patelliforme* phylogeny. While numerous studies have identified and analyzed phylogenetic relationships within the *Corispermum* genus using DNA barcode fragments (Xue and Zhang 2011; Huang et al. 2020), research on chloroplast genome phylogeny is lacking.

Chloroplast are essential organelles that are central participants in plant cells and involved in photosynthesis and carbon fixation (Hollingsworth et al. 2016; Yan et al. 2024). In most plants, the chloroplast genome has a small size, haploid nature and single-parental inheritance, which make it a good option for the analyses of nucleotide diversity and reconstructing phylogenies of closely related species (Zhang et al. 2022). In recent years, comparative and phylogenetic analyses of chloroplast genomes have proved an ideal tool for species identification, resolving phylogenetic relationships, and reconstructing the evolutionary history (Zhang et al. 2022; Yisilam et al. 2025).

In this study, we assembled and analyzed the complete chloroplast genome of *C. patelliforme* for the first time. Our aims of this study were (1) to elucidates characterize the structural features of the chloroplast genome for the *C. patelliforme*, and (2) to resolve the evolutionary relationships of *C. patelliforme*, and to provide data support for the species identification and phylogenetic relationship of *Corispermum*.

## 2. Materials

**Figure 1.**
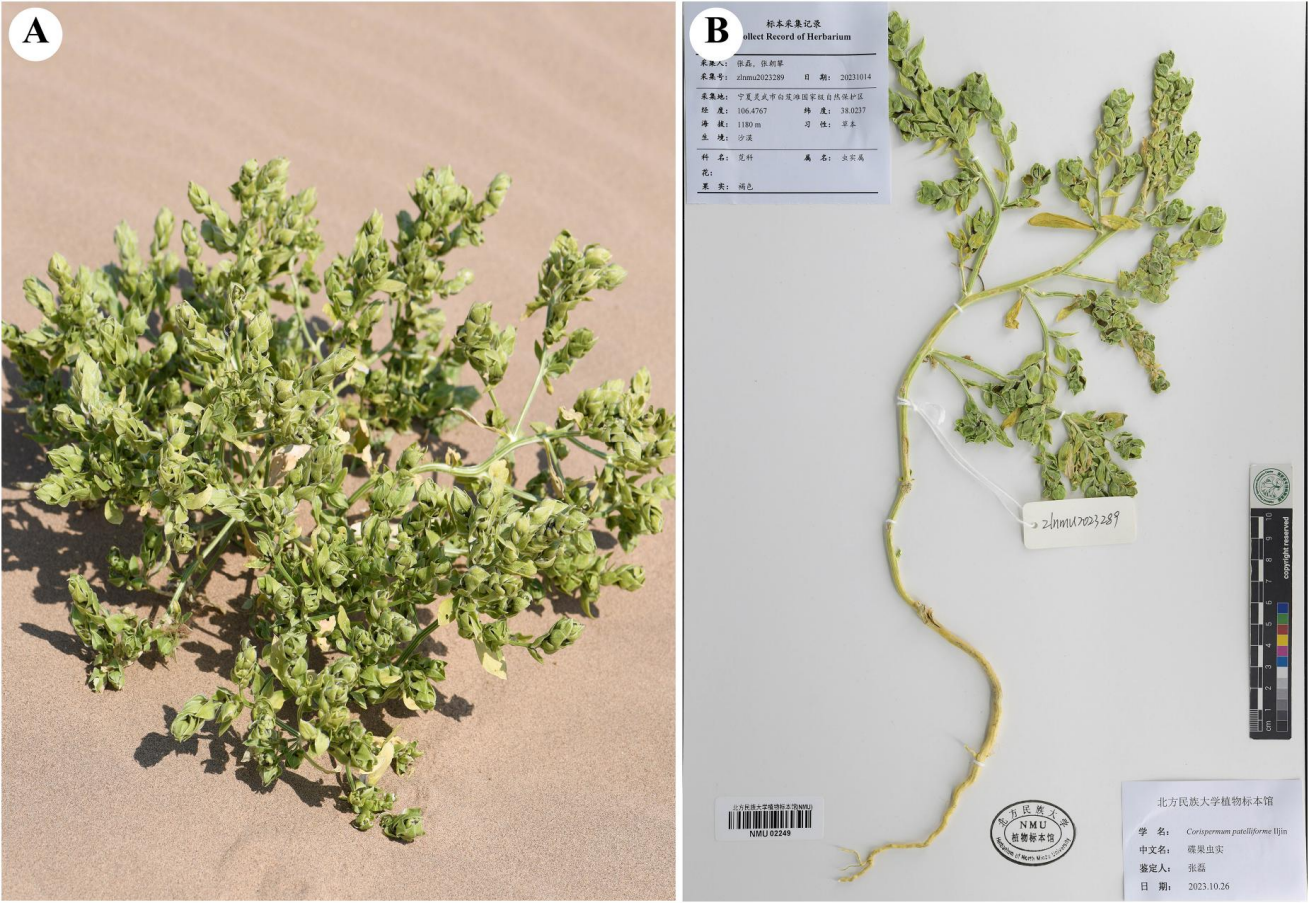
An individual plant of *C. patelliforme* (photoed by Chaopan Zhang). (B) Herbarium of *C. patelliforme* (the leaves are broad, three-veined; the fruits are disc-shaped.).

Fresh leaves of *C. patelliforme* were collected from LingWu (Yinchuan, NingXia, China; coordinates: 106.4767E, 38.0237 N) (by Chaopan Zhang: 15585866463@163.com) (Figure 1), and desiccated using silica gel. In addition, whole plant with flowers or fruits collected to prepare voucher specimens. (zhangsanshi-0319@163.com), and subsequently desiccated using silica gel for DNA extraction. A specimen was deposited at Herbarium of North Minzu University (Lei Zhang: zhangsanshi-0319@163.com) under the voucher number NMU02249.

## 3. Methods

Total genomic DNA was isolated with a modified cetyl trimethyl ammonium bromide (CTAB) method (Doyle and Doyle 1987). The NEBNext DNA Library Kit was employed to create the sequencing libraries following the manufacturer’s instructions. DNA was randomly sheared to a size of 350 bp. This library was sequenced on the Illumina NovaSeq 6000 platform with 150 bp paired-end read length. We acquired 6.2 Gb of high-quality paired-end reads for *C. patelliforme*.

For chloroplast genome assemble, the *de novo* assembly was implemented in NOVOPlasty 4.3 (Dierckxsens et al. 2017) with the specified parameters: k-mer = 39 and genome range 120,000–200,000 bp, using the complete chloroplast genome of *Corispermum declinatum* Steph. ex Steven (OR458832) as a reference. Genome annotation was executed through Plann v1.1 (Huang and Cronk 2015) with subsequent manual verification in Geneious v11.0.3 (Kearse et al. 2012). Sequencing depth analysis was quantified using Samtools (Li et al. 2009).

To further elucidate the phylogenetic placement of *C. patelliforme* in Amaranthaceae, the chloroplast genomes of 13 representative species were retrieved from NCBI GenBank to reconstruct the chloroplast genome phylogenetic tree, with *Dianthus chinensis* L. (OP136025) serving as an outgroup. All the sequences were aligned using MAFFT v.7.313 (Katoh and Standley 2013). We used RAxML v8.1.24 (Stamatakis 2014) to conduct maximum likelihood (ML) analyses with the GTR + Γ model. The optimal model (GTR+I + G) was identified using jModeltest and Bayesian inference (BI) analysis was conducted in Mrbayes v 3.2.6 (Ronquist et al. 2012). FigTree v1.4.2 (Rambaut 2012) was subsequently utilized to visualize the phylogeny.

## 4. Results

**Figure 2.**
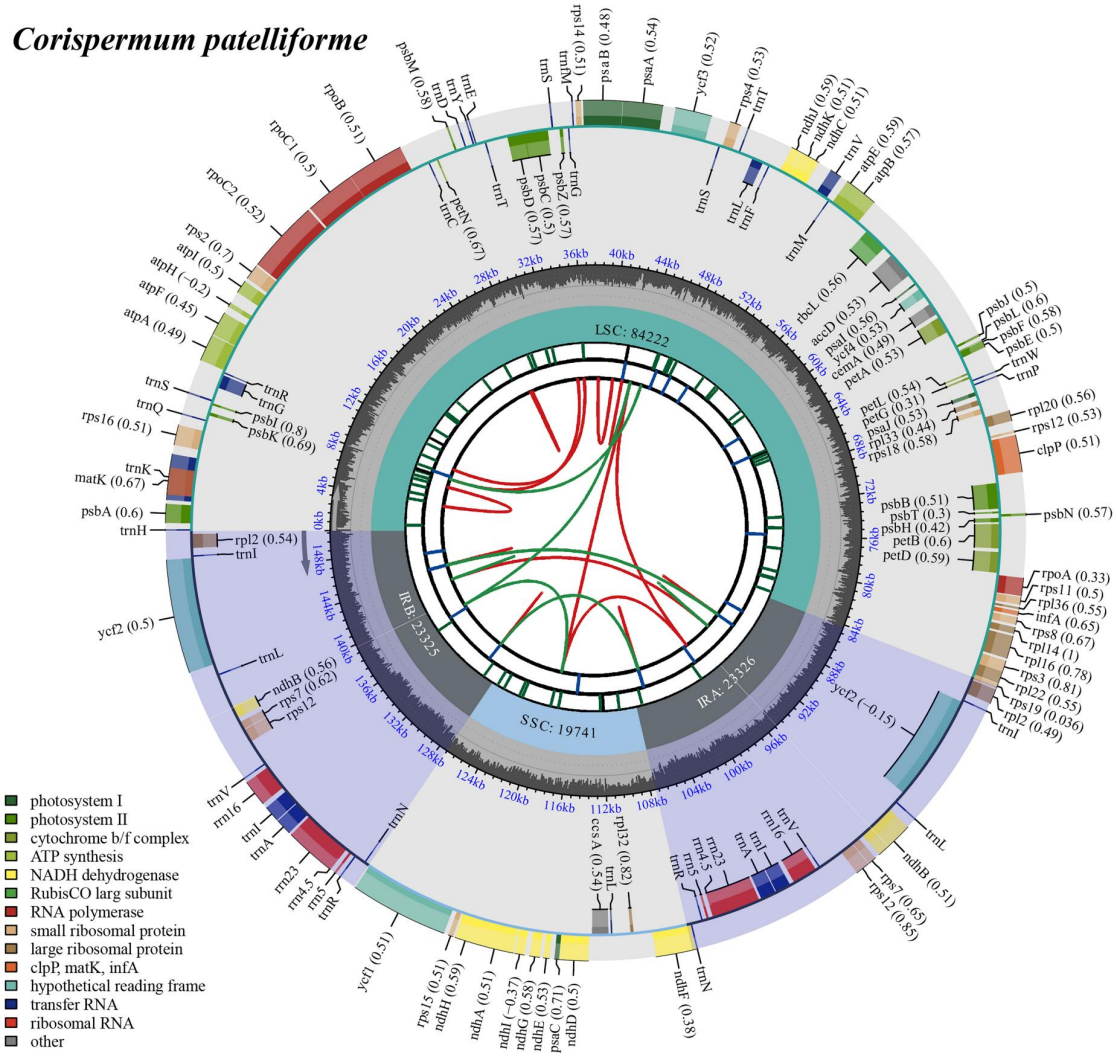
The detailed genome collinearity map of *C. patelliforme* cp genome. The species name and GC content are shown in the top left corner. The map contains six tracks in default. From the center outward, the first track shows the dispersed repeats. The dispersed repeats consist of direct and palindromic repeats, connected with red and green arcs. The second track shows the long tandem repeats as short blue bars. The third track shows the short tandem repeats or microsatellite sequences. The fourth track displays the genome length. The fifth track shows the GC content along the genome, while the sixth track sounds the genes. The gene names are followed by optional information about codon usage bias and color-coded based on their functional classification. The inner genes are transcribed clockwise, and the outer genes are transcribed anticlockwise. The functional type of the genes is shown in the bottom left corner.

**Figure 3.**
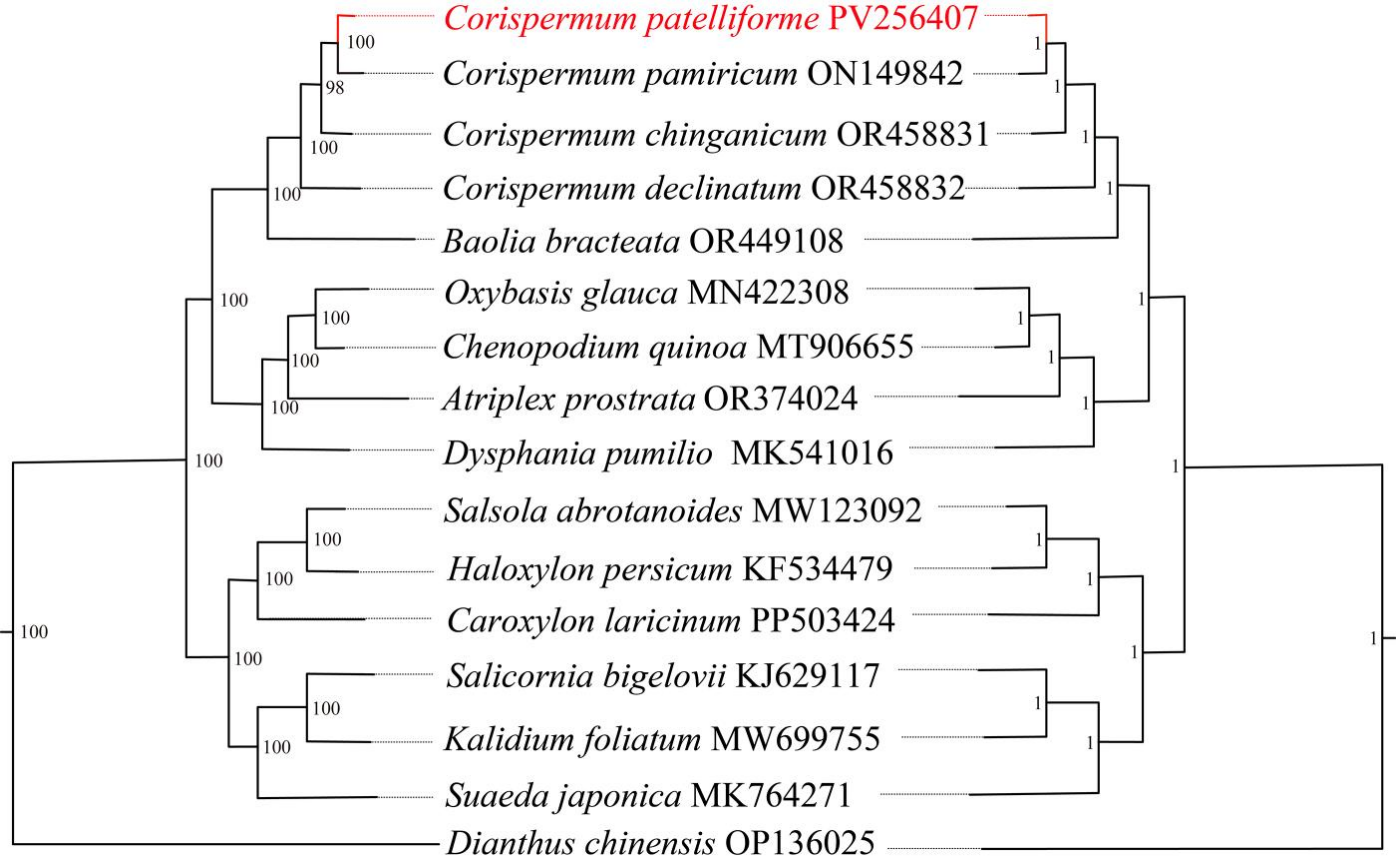
Phylogram trees based on whole chloroplast genome sequences for Amaranthaceae species with *Dianthus chinensis* as the outgroup. The left and right trees are use the maximum likelihood (ML) and Bayesian inference (BI) methods respectively. GenBank accession numbers: *Atiplex prostrata* OR374024 (Wei et al. 2023), *Baolia bracteata* OR449108 (Liu et al. 2024), *Caroxylon laricinum* PP503424 (Almerekova et al. 2024), *Chenopodium quinoa* MT906655, *Corisperum chinganicum* OR458831 (Liu S et al. 2024), *Corispermum declinatum* OR458832 (Liu et al. 2024), *Corispermum pamiricum* ON149842 (Xu et al. 2024), *Corispemum patelliforme* PV256407, *Dianthus chinensis* OP136025, *Dysphania pumilio* MK541016 (Park and Kim 2019), *Haloxylon persicum* KF534479 (Dong et al. 2016), *Kalidium foliatum* (MW699755), *Oxybasis glauca* MN422308 (Yao et al. 2019), *Salsola abrotanoides* MW123092 (Li et al. 2021), *Salicornia bigelovii* KJ629117, *Suaeda japonica* MK764271 (Kim et al. 2020).

After quality control and preprocessing, we obtained at least 4.76 gigabases (Gb) of whole-genome sequencing data. The clean reads were used to assemble high-quality chloroplast genomes through a reference-guided approach. The total chloroplast genome of *C. patelliforme* (PV256407) was 150,614 bp long, with average depths of 6821 x, 118 x, and 3205.46 x for maximal, minimal, and average, respectively (Figure S1). It exhibited a typical quadripartite structural organization, comprising a large single-copy (LSC) region of 84,222bp, two inverted repeat (IR) regions of 46,652bp each, and a small single-copy (SSC) region of 19,740bp (Figures 2 and S2). The chloroplast genome harbored 128 complete genes (Table S1), including 83 protein-coding genes (PCGs), eight ribosomal RNA genes (rRNAs), and 37 transfer RNA (tRNA) genes. Most genes were present in a single copy, while 16 genes were duplicated, encompassing four rRNAs (*rrn*16, *rrn*23, *rrn*4.5, and *rrn*5), seven tRNAs (*trn*I-CAU, *trn*L-CAA, *trn*V-GAC, *trn*I-GAU, *trn*A-UGC, *trn*R-ACG, *trn*N-GUU), and five PCGs (*ndh*B, *rpl*2, *rps*7, *rps*12, *ycf*2). Additionally, the chloroplast genome contained 10 cis-splicing genes (Figure S3) and 1 trans-splicing gene (Figure S4). The overall GC content of the chloroplast DNA was 36.5%, with the LSC, SSC, and IR regions having corresponding values of 34.2%, 29.8%, and 43.2%, respectively. Both ML and BI trees confirmed the placement of *C. patelliforme* within the Amaranthaceae family (Figures 3 and S5). In these trees, the 15 Amaranthaceae species formed a monophyletic group, dividing into four main clades with strong support base score (BS), percentage point (PP) = 100%, 1 (Figures 3 and S5). All *Corispermum* species together into a subclade and *Baolia bracteata* H. W. Kung & G. L. Chu (OR449108) is placed as sister to *Corispermum.* While, *C. patelliforme* and *C. pamiricum* Iljin (ON149842) formed a monophyletic group (BS, PP = 100%, 1), suggesting a close relationship between the two species.

## 5. Discussion and conclusion

Previous research studies have demonstrated that angiosperm chloroplast genomes typically exhibit high conservation in terms of genomic architecture, gene arrangement, and content, characterized by a quadripartite organization comprising two inverted repeat (IR) regions that separate the large single-copy (LSC) and small single-copy (SSC) regions (Daniell et al. 2016; Yang et al. 2022). Most chloroplasts are circular and ranged from 107 kb in Pinaceae to 170 kb in Geraniaceae (Wu et al. 2020), with 110 to 130 different genes. In this study, the complete cp genome of *C. patelliforme* was assembled with a total sequence length of 150,614 bp, at the larger end of the spectrum for seed plants organelle genomes. In addition, angiosperm chloroplast genomes generally maintain an average GC content of 35%, the *C. patelliforme* chloroplast demonstrates a marginally elevated GC composition of 36.5%, exceeding the typical angiosperm average.

The cp genomes have become a critical focus in molecular biological studies and have shown substantial power in solving phylogenetic relationships among angiosperms (Yang et al. 2022; Ran et al. 2024). In this study, a phylogenetic tree was developed utilizing the ML and BI methods with *D. chinensis* as the outgroup. The tree revealed that the cp genomes of Amaranthaceae species dividing into four main clades with strong support, with *C. patelliforme* and *C. pamiricum* shares the closest relationship, formed a monophyletic group, which is in agreement with the phylogenetic tree based on the ITS sequences (Li et al. 2021). In addition, *Baolia bracteata*, which is nested within *Corispermum*, is identified as the sister group to *Corispermum*, suggesting that *Baolia* represents a non-monophyletic genus. These results are consistent with previous studies on the phylogenetic relationships within Amaranthaceae (Li et al. 2021; Xu et al. 2024; Vanessa et al. 2025).

In this study, we assembled the chloroplast genomes of *C. patelliforme* for the first time. The result revealing a 150,614 bp circular structure with typical quadripartite organization, and is closely related to *C. pamiricum*. Further investigation of *C. patelliforme* is necessary, including additional studies at the population level and genome analysis. Only through thorough analysis can understand the structural variation of the chloroplast genome of *C. patelliforme*. In addition, expanding the collection of *Corispermum* cp genomes will provide deeper insights into the evolution of this important genus.

## Supplementary Material

Supplementary File.docx

## Data Availability

The sequenced data supporting the findings of this study are openly available in NCBI (https://www.ncbi.nlm.nih.gov/) under the accession no. PV256407. The associated BioProject, SRA and Bio-Sample numbers are PRJNA1232922, SRR32607550 and SAMN47252372, respectively.
