# Supplementary material for: The complete chloroplast genome of Corispermum patelliforme (Amaranthaceae): genome characterization and phylogenetic consideration
Source: Mitochondrial DNA B Resour. 2025 Sep 18;10(10):976–80. doi: 10.1080/23802359.2025.2561080 (PMC12447465; doi:10.1080/23802359.2025.2561080)
Supplement: Supplementary File.docx [file TMDN_A_2561080_SM8653.docx]

The complete chloroplast genome of *Corispermum patelliforme* (Amaranthaceae): genome characterization and phylogenetic consideration

Zhen Yang^1^, Jinju Zhao^2^, Wenqin Li^1^,Yangjie Chen^1^, Chaopan Zhang^2^, Hongqiang Lin^3*^

^1^Department of Brewing Engineering, Moutai Institute, Luban Ave, Renhuai 564507, Guizhou, P. R. China

^2^School of Biological Science & Engineering, North Minzu University, Yinchuan, Ningxia, P. R. China

^3^Sichuan Wolong National Natural Reserve Administration Bureau, Wenchuan 623006, Sichuan, P. R. China

***Corresponding author**: Hongqiang Lin, 13348986271@163.com

**ORCID**: Hongqiang Lin; https://orcid.org/0000-0002-0659-9734

Table S1 Genes present in the *Corispermum patelliforme* cp genome

| Group of genes | Name of genes |
| --- | --- |
| Subunits of ATP synthase | *atp*A, *atp*B, *atp*E, *atp*F*, *atp*H, *atp*I |
| Subunits of NADH-dehydrogenase | *ndh*A*, *ndh*B* (×2), *ndh*C, *ndh*D, *ndh*E, *ndh*F, *ndh*G, *ndh*H, *ndh*I, *ndh*J, *ndh*K |
| Subunits of cytochrome b/f complex | *pet*A, *pet*B*, *pet*D*, *pet*G, *pet*L, *pet*N |
| Subunits of photosystem I | *psa*A, *psa*B, *psa*C, *psa*I, *psa*J |
| Subunits of photosystem II | *psb*A, *psb*B, *psb*C, *psb*D, *psb*E, *psb*F, *psb*H, *psb*I, *psb*J, *psb*K, *psb*L, *psb*M, *psb*N, *psb*T, *psb*Z |
| Large subunit of ribosome | *rpl*14, *rpl*16*, *rpl*20, *rpl*23, *rpl*32, *rpl*33, *rpl*36, *rpl*2** (×2) |
| Small subunit of ribosome | *rps*2, *rps*3, *rps*4, *rps*7 (×2), *rps*8, *rps*11, *rps*14, *rps*15, *rps*18, *rps*19, *rps*12** (×2), *rps*16* |
| DNA dependent RNA polymerase | *rpo*A, *rpo*B, *rpo*C1*, *rpo*C2 |
| Subunit of rubisco | *rbc*L |
| c-type cytochrom synthesis gene | *ccs*A |
| Envelop membrane protein | *cem*A |
| Maturase | *mat*K |
| Protease | *clp*P** |
| Subunit of Acetyl-CoA-carboxylase | *acc*D |
| Translational initiation factor | *infA* |
| Conserved open reading frames | *ycf*1, *ycf2* (×2), ycf3**, *ycf*4 |
| trnA | *trn*H-GUG, *trn*K-UUU*, *trn*Q-UUG, *trn*S-GCU, *trn*G-GCC*, *trn*R-UCU, *trn*C-GCA, *trn*D-GUC, *trn*Y-GUA, *trn*E-UUC, *trn*T-GGU, *trn*S-UGA, *trn*G-UCC, *trn*M-CAU, *trn*S-GGA, *trn*T-UGU, *trn*L-UAA*, *trn*F-GAA, *trn*V-UAC*, *trn*M-CAU, *trn*W-CCA, *trn*P-UGG, *trn*I-CAU* (×2), *trn*L-CAA (×2), *trn*V-GAC (×2), *trn*I-GAU (×2), *trn*A-UGC* (×2), *trn*R-ACG (×2), *trn*N-GUU (×2), *trn*L-UAG, |
| rRNA | *rrn*4.5 (×2), *rrn*5 (×2), *rrn*16 (×2), *rrn*23 (×2) |
| Genes with one or two introns are indicated by one (*) or two asterisks (**), respectively. Two gene copies are folowed by the (×2) symbol. | |


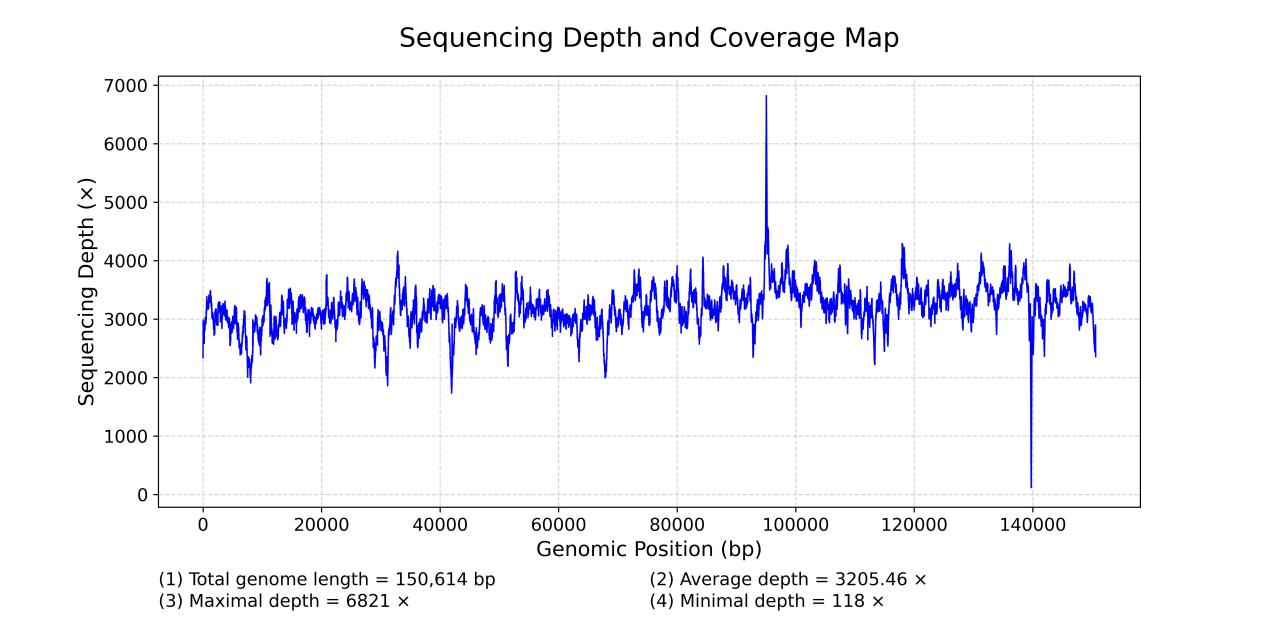


Figure S1 Coverage depth distribution of the *C. patelliforme*.


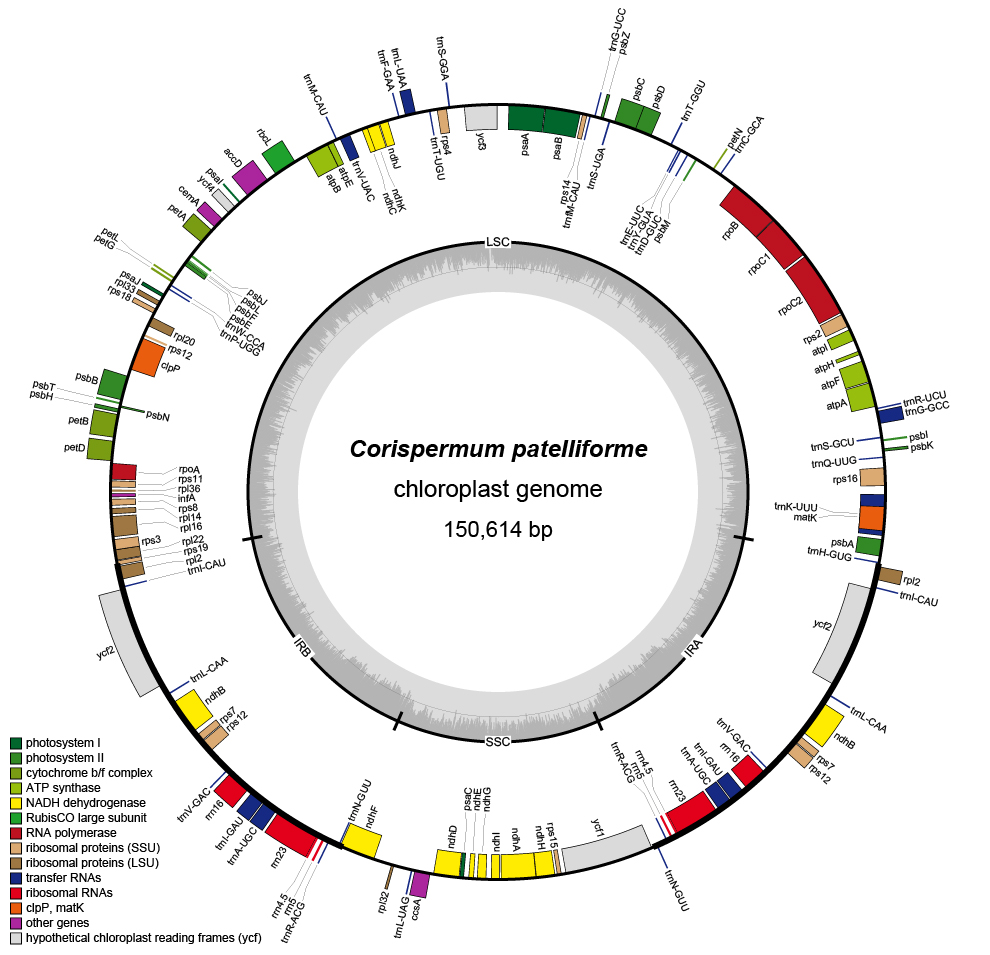


Figure S2 The detailed genome map of *C. patelliforme* cp genome. GC content (light gray) is shown in the inside track. Gene models including protein-coding genes, tRNA genes and rRNA genes are shown with various colored boxes in the outer track.


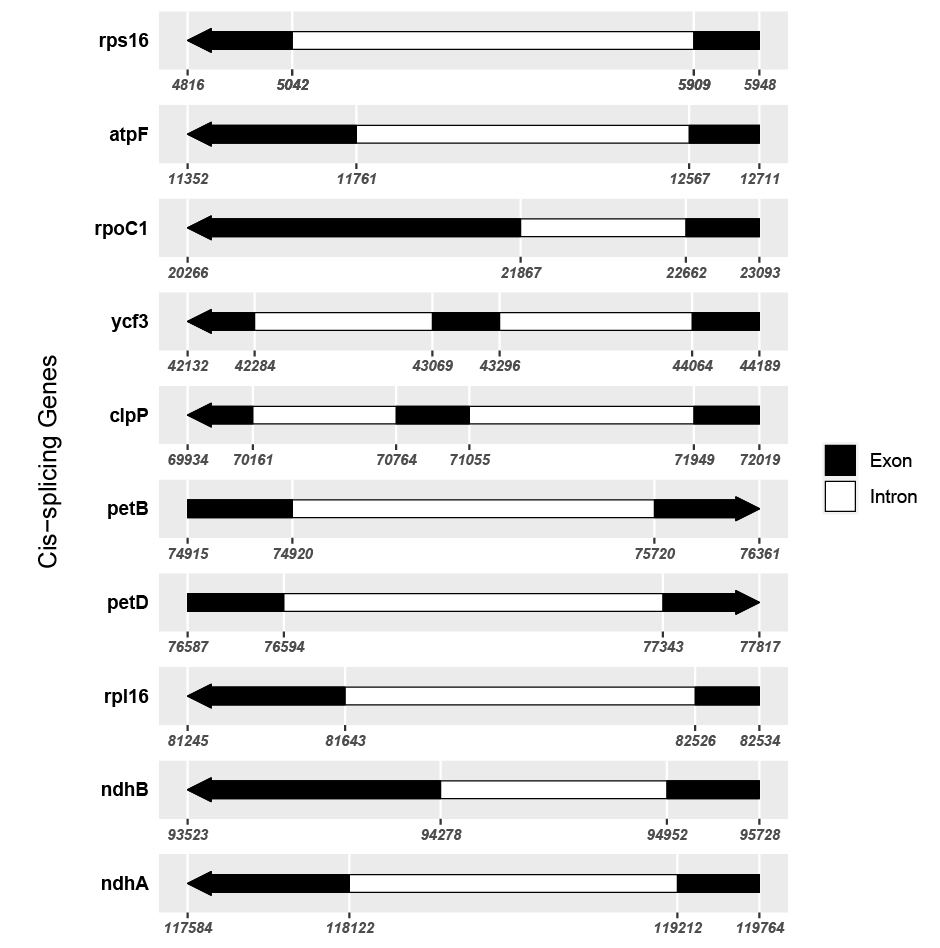


Figure S3 Structure of Cis-splicing genes in the *C. patelliforme*.


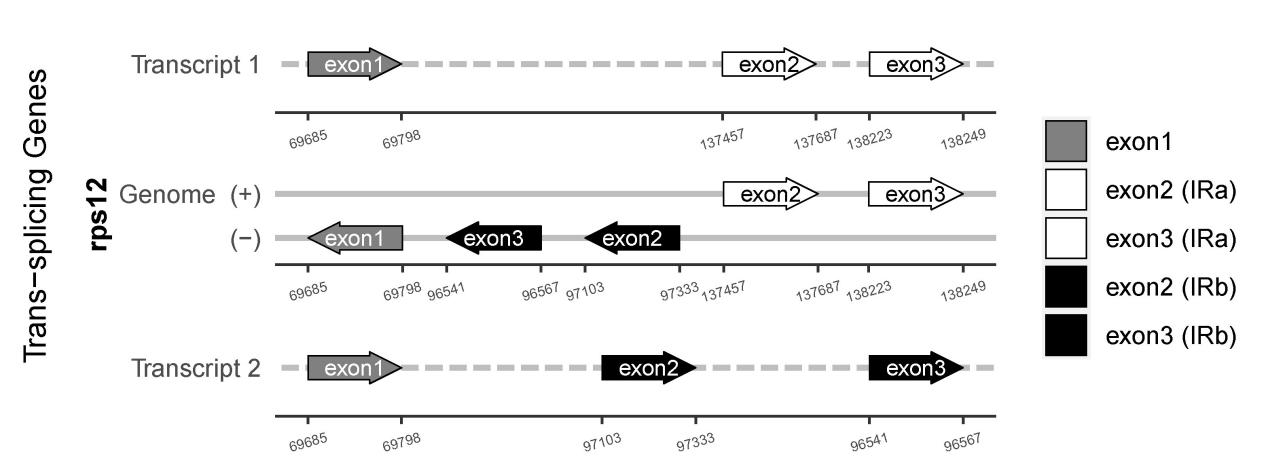


Figure S4 Structure of trans-splicing genes in the *C. patelliforme*.


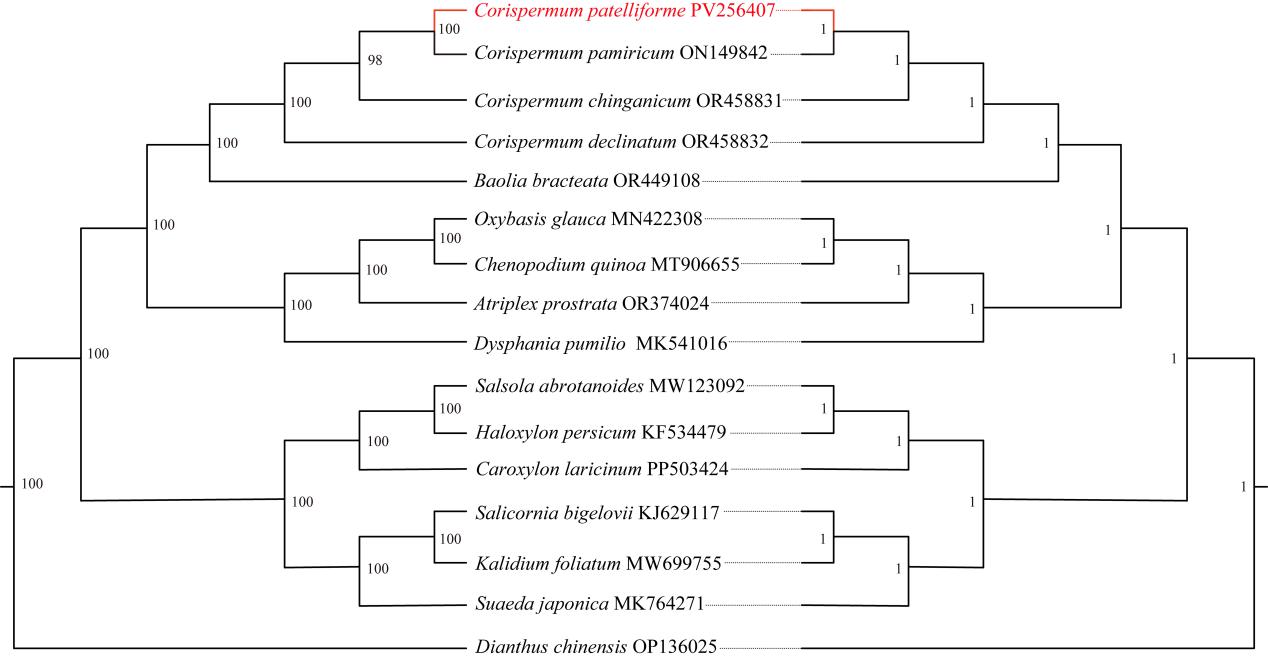


Figure S5 Cladogram trees based on whole chloroplast genome sequences for Amaranthaceae species with *Dianthus chinensis* as the outgroup. The left and right trees are use the Maximum Likelihood (ML) and Bayesian inference (BI) methods respectively.
